# Comparative physiological and transcriptome analysis between potassium-deficiency tolerant and sensitive sweetpotato genotypes in response to potassium-deficiency stress

**DOI:** 10.1186/s12864-023-09939-5

**Published:** 2024-01-15

**Authors:** Rong Jin, Mengxiao Yan, Guanghua Li, Ming Liu, Peng Zhao, Zhe Zhang, Qiangqiang Zhang, Xiaoya Zhu, Jing Wang, Yongchao Yu, Aijun Zhang, Jun Yang, Zhonghou Tang

**Affiliations:** 1 Xuzhou Sweetpotato Research Center, Xuzhou Institute of Agricultural Sciences, Xuzhou, Jiangsu China; 2 Key Laboratory of Sweetpotato Biology and Genetic Breeding, Ministry of Agriculture, National Agricultural Experimental Station for Soil Quality, Xuzhou, Jiangsu China; 3 Jiangsu Key Laboratory of Phylogenomics and Comparative Genomics, School of Life Sciences, Jiangsu Normal University, Xuzhou, 221116 Jiangsu China; 4 sishui lifeng food products Co., Ltd, Jining, China; 5 Shanghai Key Laboratory of Plant Functional Genomics and Resources, Shanghai Chenshan Plant Science Research Center, Chinese Academy of Sciences, Shanghai Chenshan Botanical Garden, Shanghai, 201602 China; 6 Sishui County Agriculture and Rural Bureau, Jining, China

**Keywords:** Sweetpotato, Genotype, K^+^-deficiency, RNA-seq, Differentially expressed genes (DEGs)

## Abstract

**Background:**

Sweetpotato is a typical ‘‘potassium (K^+^) favoring’’ food crop, which root differentiation process needs a large supply of potassium fertilizer and determine the final root yield. To further understand the regulatory network of the response to low potassium stress, here we analyze physiological and biochemical characteristics, and investigated root transcriptional changes in two sweetpotato genotypes, namely, - K tolerant “Xu32” and - K susceptible“NZ1”.

**Result:**

We found Xu32 had the higher capability of K^+^ absorption than NZ1 with better growth performance, higher net photosynthetic rate and higher chlorophyll contents under low potassium stress, and identified 889 differentially expressed genes (DEGs) in Xu32, 634 DEGs in NZ1, 256 common DEGs in both Xu32 and NZ1. The Gene Ontology (GO) term in molecular function enrichment analysis revealed that the DEGs under low K^+^ stress are predominately involved in catalytic activity, binding, transporter activity and antioxidant activity. Moreover, the more numbers of identified DEGs in Xu32 than that in NZ1 responded to K^+^-deficiency belong to the process of photosynthesis, carbohydrate metabolism, ion transport, hormone signaling, stress-related and antioxidant system may result in different ability to K^+^-deficiency tolerance. The unique genes in Xu32 may make a great contribution to enhance low K^+^ tolerance, and provide useful information for the molecular regulation mechanism of K^+^-deficiency tolerance in sweetpotato.

**Conclusions:**

The common and distinct expression pattern between the two sweetpotato genotypes illuminate a complex mechanism response to low potassium exist in sweetpotato. The study provides some candidate genes, which can be used in sweetpotato breeding program for improving low potassium stress tolerance.

**Supplementary Information:**

The online version contains supplementary material available at 10.1186/s12864-023-09939-5.

## Background

Potassium (K^+^), as one of the most essential macronutrients, plays important roles in plants and contributes greatly to enzyme activation, protein synthesis, photosynthesis, osmotic pressure, and cell extension. K^+^ deficiency in the field seriously influences agriculture through a series of negative impacts, such as growth inhibition, impaired nitrogen uptake, increased pathogen susceptibility, osmotic imbalance, and finally crop failure [1]. Sweetpotato (*Ipomoea batatas* [L.] Lam), a typical ‘‘K^+^ favoring’’ food crop, plays a critical role in both food security and bio-industries. However, the K^+^ deficiency of soil in southern China seriously limits sweetpotato productivity and quality in this region [2].

The dry matter yield and biomass productivity are closely related to the soil potassium supply in sweetpotato. Its root differentiation process requires a large supply of potassium fertilizer and determines the final root yield, which has a decisive effect on market supply and economic benefits [3]. Hence, understanding how sweetpotato responds to low-K^+^ stress is valuable because the complex molecular mechanism and regulatory network have not been fully elucidated.

Plants resist low-K^+^ stress at the physiological level through regulating the cellular and tissue homeostasis of K^+^. K^+^ transporters and K^+^ channel proteins function in potassium uptake. There are five K^+^ transporter gene families, including the HAK/KUP/KT family, Trk/HKT family, KEA family, CHX family, and Shake K^+^ channel family in plants [4, 5]. The Shaker family in Arabidopsis contains 9 members, which have been well studied. The inward rectifying channel AKT1 as the first reported gene, was strongly expressed by salt and low K^+^ stress [6]. It is reported that AKT1 and the high-affinity K^+^ transporter HAK5 dominate above 95% of K^+^ absolution [7]. Our previous study identified 22 HAK/KUP/KT genes and nine shake K^+^ channel genes in sweetpotato and found that *IbAKT1*-overexpressing transgenic roots could absorb more K^+^ under K^+^-deficiency stress [8, 9]. *AtKCl* does not possess K^+^ channel activity, but it can balance K^+^ uptake/leakage to modulate AKT1-mediated low K^+^ responses [10]. K^+^ outward rectifying channel *SKOR* is involved in mediating long-distance K^+^ transport from roots to shoots [11]. *AKT2* mediate dual-directional K^+^ transport with weak voltage-dependency and realize long-distance transportation of K^+^ in phloem [12]. Inward K^+^ channel *SPIK* expressed in pollen tubes and functions in the viability of pollen tubes [13].The inward potassium ion channels *KAT1*, *KAT2*, and an outward potassium ion channel *GORK* are mainly expressed in guard cells and regulate osmotic potential and stomatal movement [14]. In additional, the activity of potassium ion channels in plants is regulated by various proteins, such as protein kinases and G proteins [15, 16]. However, additional in-depth studies on low-K^+^ tolerance mechanism have not been reported.

Transcriptome technology as a convenient tool can rapid distinguishes differentially expressed genes (DEGs) under a variety of environmental conditions. Some important genes encoding kinases, transcription factors, carbohydrates, or involved in the signal transduction pathway including second messenger, reactive oxygen species (ROS), plant hormones during the plants responses to K-deficiency were identified via transcriptomic analyses in many plants, including rice, wheat, apple and banana [17–20].

To understand the molecular and physiological mechanism of sweetpotato resistance to low-K^+^ stress, Xu32 (a low-K^+^ tolerant genotype) and NZ1 (a low-K^+^ sensitive genotype) were screened from 31 sweetpotato materials according to their K^+^ utilization [2]. In this study, the physiology and biochemical indexes were compared between the two sweetpotato genotypes, and RNA sequencing (RNA-seq) was performed to explore differences in the responses in transcriptome profiles by characterizing the temporal patterns of expression and gene regulation under low-K^+^ conditions.

## Results

### Physiological performance analysis of two sweetpotato genotypes in response to K^+^deficiency

**Fig. 1:**
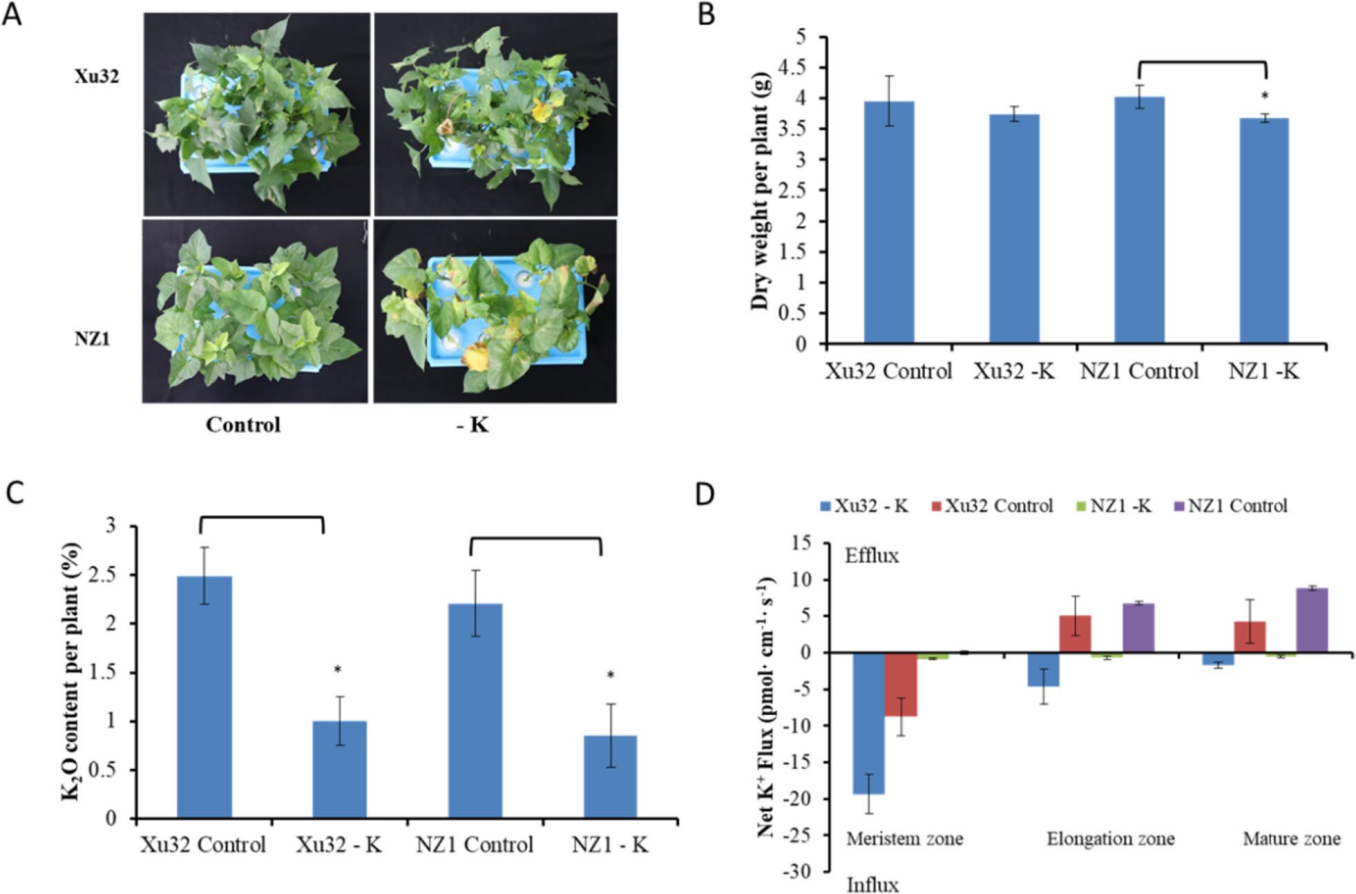
Different sensitivity to K^+^ deficiency between K^+^-stress-tolerant sweetpotato cultivar Xu32 and K^+^-stress-sensitive sweetpotato cultivar NZ1.Phenotypes (**A**), dry weight (**B**), potassium content (**C**) and net K^+^ flux (**D**) of hydroponic-cultured sweetpotato seedlings under normal (Control) and K^+^-deficient conditions (-K) for two weeks. Date are means ± SE (*n* = 3). For (B) and (C), difference between mean values of -K and control were compared using t-tests (^*^ *P* < 0.05)

**Fig. 2:**
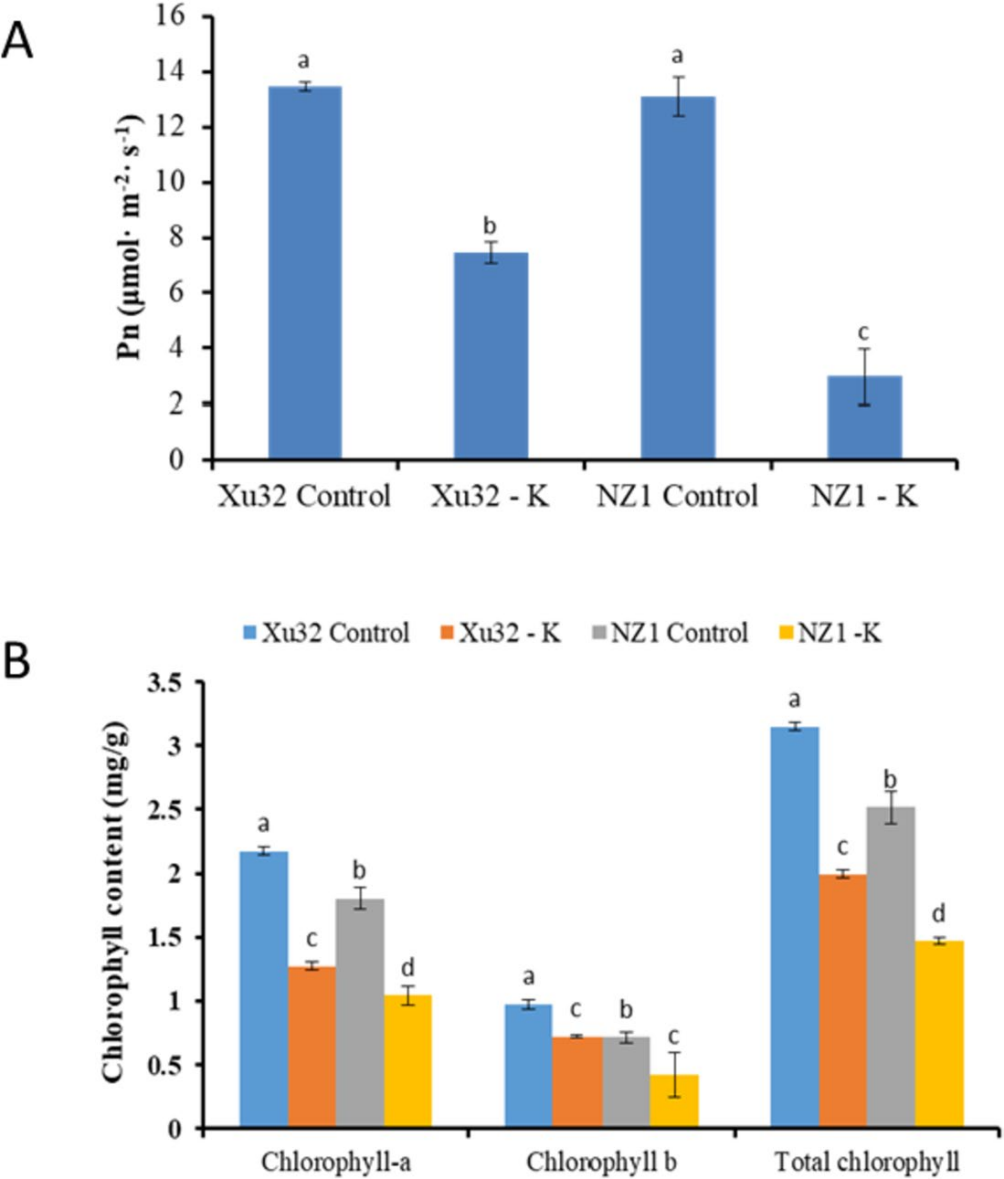
K^+^ deficiency affects the photosynthetic system of two different K^+^-sensitive sweetpotato cultivars, Xu32 and NZ1.The net photosynthetic rate (Pn) (**A**) and chlorophyll contents (**B**) of hydroponic-cultured sweetpotato seedlings under normal (Control) and K^+^-deficient conditions (-K) for two weeks. Date are means ± SE (*n* = 3). Different letters (a-d) denote significant differences at *P* < 0.05

Significant symptoms developed in the two sweetpotato genotypes between the control and the low-K^+^ treatments. Although both genotypes did not grow well under the low-K^+^ treatment, NZ1 exhibited poorer performance than Xu32. Some leaves of NZ1 become chlorosis and necrosis, while leaves of Xu32 still remained green (Fig. 1A). Hence, the average dry weight per plant was obviously reduced in NZ1 under low-K^+^ conditions, whereas there was no significant reduction in Xu32 (Fig. 1B). K^+^-deficiency stress caused a significant reduction of the Pn and chlorophyll content in both genotypes. NZ1 was much more affected than Xu32, with NZ1 and Xu32 showing 41.70% and 36.63% reductions in Pn, respectively, and 77.21% and 44.71% reductions in total chlorophyll content, respectively (Fig. 2A and B). The total soluble sugar content exhibited a slight increase in Xu32 (0.87%) and a slight decrease in NZ1 (-4.40%) (Fig. S1).

Although there was little difference in K^+^ concentrations between the two genotypes under low-K^+^ treatment (Fig. 1C), the K^+^ influx rate in the roots differed between the two genotypes. The K^+^ influx rate of the meristem zone in Xu32 was markedly higher than that in NZ1 roots under normal conditions (Fig. 1D). The K^+^ efflux rates of the elongation zone and mature zone in Xu32 were 5.07 pmol cm^−2^ s^−1^ and 4.28 pmol cm^−2^ s^−1^, respectively, while the rates in NZ1 were 6.78 pmol cm^−2^ s^−1^ and 8.81 pmol cm^−2^ s^−1^, respectively. K^+^-deficiency stress enhanced K^+^ efflux in both sweetpotato genotypes. After 15 days of low-K^+^ treatment, the K^+^ effluxes in the elongation zone and mature zone of Xu32 were lower than those in NZ1, although the differences were not significant. The K^+^ efflux leakage rate was 9.74 pmol cm^−2^ s^−1^ in the elongation zone and 5.99 pmol cm^−2^ s^−1^ in the mature zone of Xu32, compared with 7.46 pmol cm^−2^ s^−1^ in the elongation zone and 9.35 pmol cm^−2^ s^−1^ in the mature zone of NZ1. K^+^ influx was still observed in the meristem zones of both genotypes, and 22-fold higher K^+^ influx was observed in the meristem zone of Xu32 (19.34 pmol cm^−2^ s^−1^) than in that of NZ1 (0.86 pmol cm^−2^ s^−1^).

**Fig. 3:**
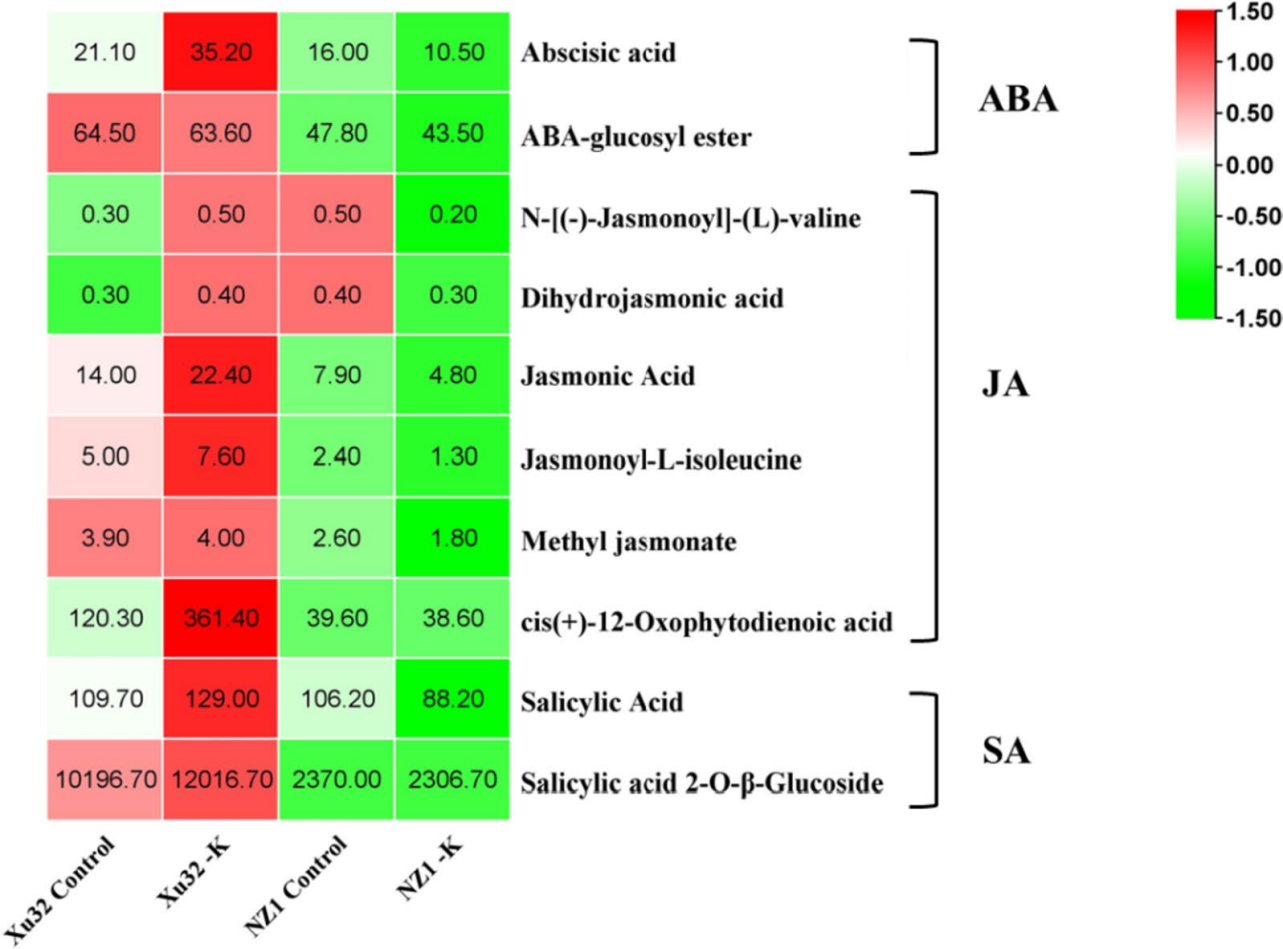
Heatmap of stress-related hormone contents in two different K^+^-sensitive sweetpotato cultivars, Xu32 and NZ1, under normal (Control) and K^+^-deficient conditions (-K) for 2 weeks. The tested materials can be classified into three hormones, namely abscisic acid (ABA), jasmonic acid (JA), and salicylic acid (SA). Data shown are the means ± standard error (SE) (*n* = 3). The color scale varies from green to red, indicating relatively low and high hormone contents, respectively

Furthermore, the contents of stress-related hormones were measured. A total of 13 materials classified into ABA, JA, and SA were identified. The opposite change trends of the hormones were found between Xu32 and NZ1. After low-K^+^ stress treatment, the contents of ABA increased by 40% in Xu32, whereas they decreased by 34.4% in NZ1; the content of its related metabolites ABA-glucosyl ester remained almost unchanged in Xu32, whereas decreased 9.0% in NZ1; the contents of JA and its related metabolites N-[(-)-Jasmonoyl]-(L)-valine, dihydrojasmonic acid, jasmonoyl-L-isoleucine, methyl jasmonate, cis(+)-12-oxphytodienoic acid increased by 37.5%, 40.0%, 25.0%, 34.2% and 66.7% in Xu32, respectively, whereas they decreased by 39.2%, 60.0%, 25.0%, 45.8% and 2.5% In NZ1, respectively; the content of SA and its related metabolites salicylic acid-O-β-glucoside increased by 15.0% and 15.1% in Xu32, respectively, whereas they decreased by 16.9% and 2.6% In NZ1, respectively (Fig. 3).

### RNA-seq data analysis and qRT-PCR validation

Two cDNA libraries (Xu32 and NZ1) were established under normal and low-K^+^ conditions. A total of 113.13 Mb and 99.31 Mb raw reads were generated using Illumina HiSeq™ technology for the Xu32 and NZ1 libraries, respectively. All of the raw reads were deposited in the NCBI SRA database (accession number PRJNA1013090). After removing the low-quality reads and trimming the adapter sequences, 109.03 Mb and 97.63 Mb clean reads were generated for the Xu32 and NZ1 libraries, respectively. In total, 73.890–83.11 Mb clean reads were successfully aligned to the sweetpotato reference genome (Table 1).

**Table 1:** Summary of the sequencing and assembly statistic for sweetpotato

Description	Xu32	NZ1
**Before triming**		
Raw Reads (*106)	111.13	99.31
Clean Reads	109,035,768	97634085.33
Clean bases(Gb)	16.35	14.65
**After triming**		
GC(%)	47.38	47.29
Q20(%)	97.44	97.57
**Assembled data**		
Mapped Reads	83117965.95	73899239.19
Mapped percentage(%)	76.23	75.69

**Fig. 4:**
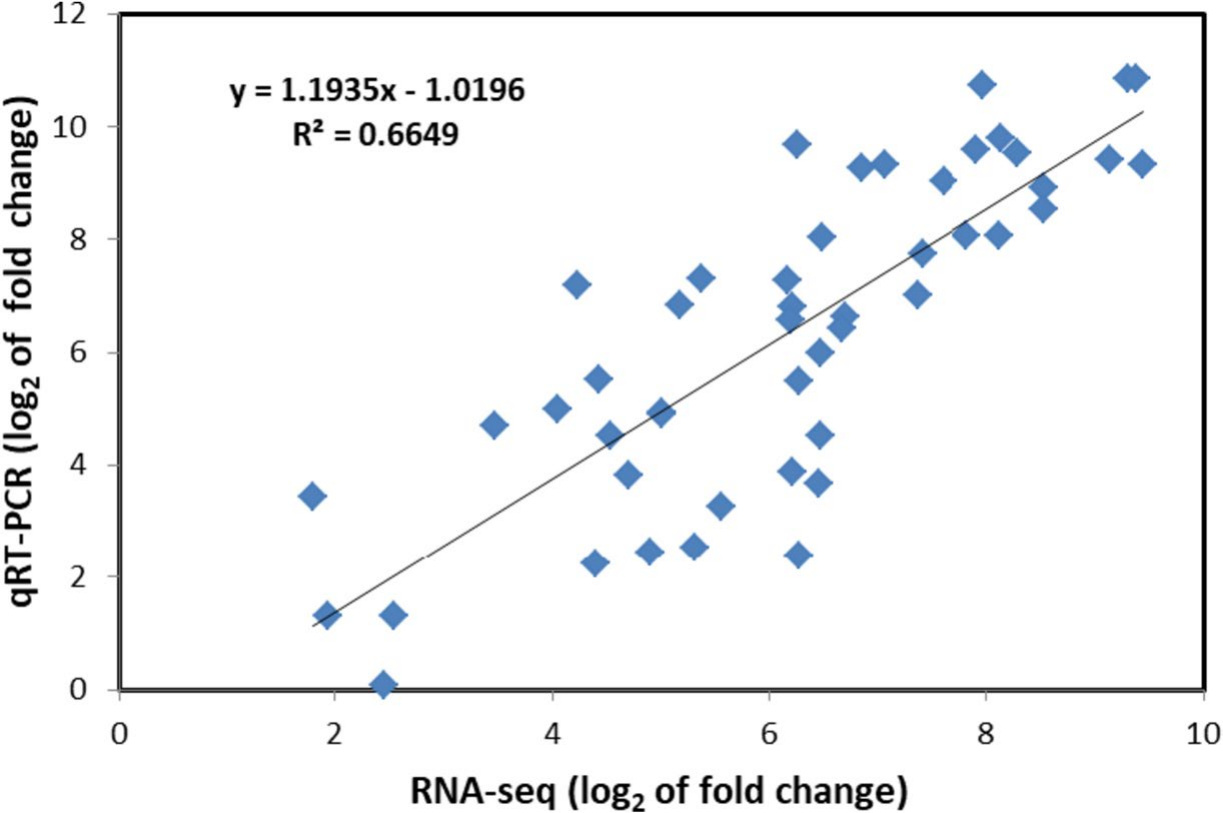
Correlation analysis of the relative expression of randomly selected common differentially expressed genes (DEGs) in Xu32 and NZ1 under normal and K^+^-deficiency (-K) treatment obtained from quantitative real-time polymerase chain reaction (qRT-PCR) and RNA sequencing (RNA-seq) data. The log2 ratio values of relative expression are between normal and -K treatment samples

Twelve randomly selected common DEGs in Xu32 and NZ1 were subjected to qRT-PCR to verify the accuracy of the RNA-seq results (Fig. S2). Correlation analysis of the relative expression between qRT-PCR and RNA-seq was performed, as shown in Fig. 4. The qRT-PCR results revealed that the gene expression trends were significantly correlated with those obtained from the RNA-seq data (r^2^ = 0.6649, Fig. 4), indicating that the RNA-seq results were reliable.

### DEG analysis in two sweetpotato genotypes in response to K^+^ deficiency

**Fig. 5:**
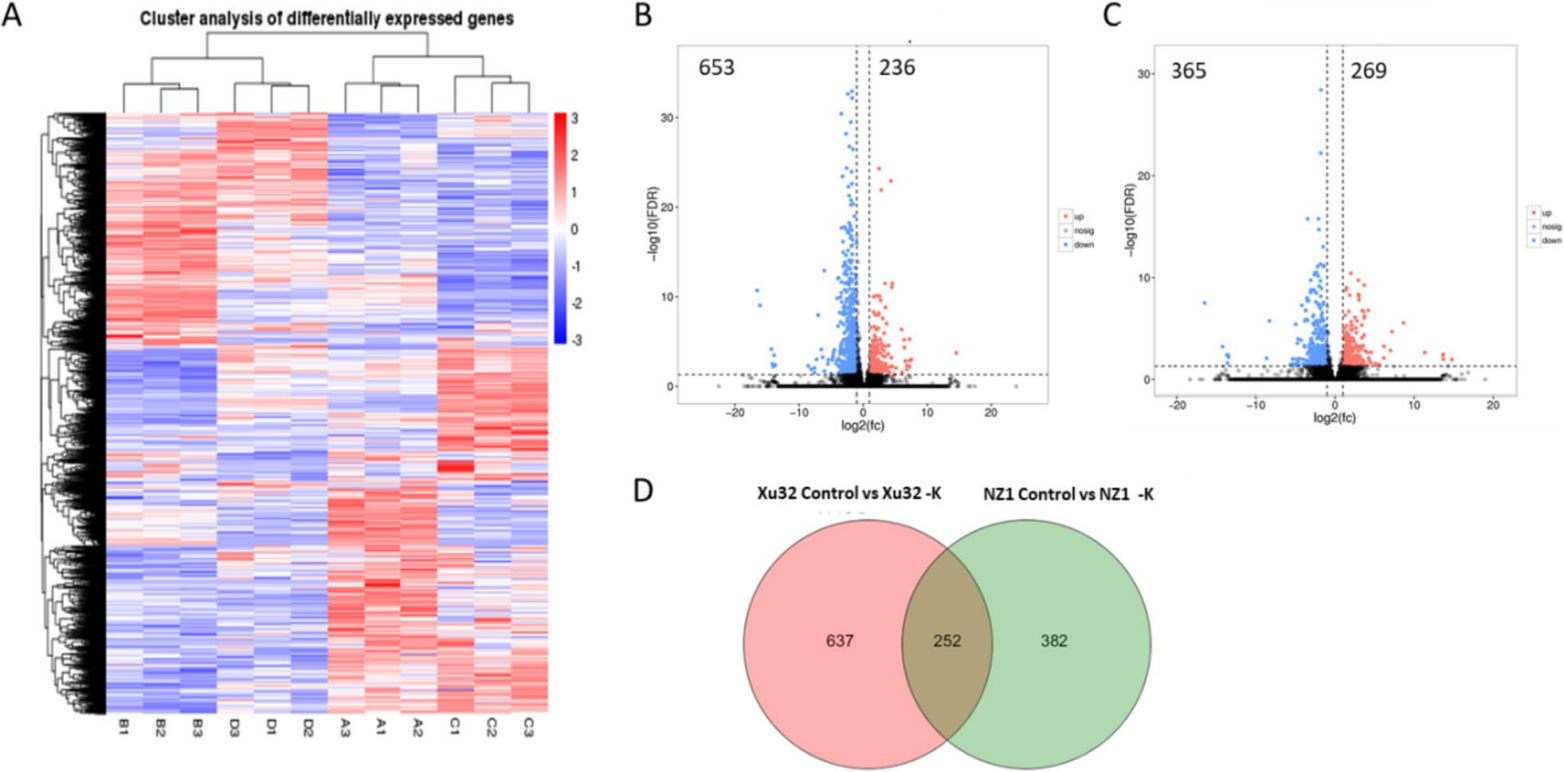
Profiles of gene expression in two different K^+^-sensitive sweetpotato cultivars, Xu32 and NZ1, exposed to K^+^-deficiency stress. **A** Hierarchical cluster analysis of differentially expressed genes (DEGs) in Xu32 and NZ1 during normal and K^+^-deficiency (-K) treatment. **B**–**C** Volcano plots showing the upregulated and downregulated genes between the normal and -K treatment in Xu32 and NZ1, respectively. **D** Venn diagram showing the overlaps among DEGs in Xu32 and NZ1 under K^+^-deficiency stress treatment

The gene expression abundance was affected under K^+^-deficiency stress in the two genotypes. The DEGs were identified with a *P* value-adjusted < 0.005 and log2 value > 1 based on pairwise comparisons between the control and low-K^+^ treatment for each genotype (Fig. 5). A total of 889 and 634 DEGs were identified in the comparisons of Xu32 Control versus Xu32 -K and NZ1 Control versus NZ1 -K, respectively, which indicated significant differences in the gene expression profiles between the control and potassium-deficiency stress groups. Notably, more DEGs were detected in Xu32 than in NZ1, suggesting that tolerance to K^+^ deficiency could influence the expression of an increased number of genes. Additionally, the number of downregulated genes was higher in Xu32 than in NZ1, but the number of upregulated genes was similar in both genotypes. There were 236 upregulated DEGs and 653 downregulated DEGs in Xu32, while there were 269 upregulated DEGs and 365 downregulated DEGs in NZ1 (Fig. 5B & C). Among these DEGs, there were 256 common DEGs in Xu32 and NZ1 under K^+^-deficiency stress.

To identify DEGs of the two sweetpotato genotypes under K^+^ deficiency, the functional annotation of GO terms was performed (Fig. S1). In Xu32, the GO term single-organism process in the biological process category was the most highly represented, followed by metabolic process, cellular process, and response to stimulus. The GO term catalytic activity in the molecular function category was the most significantly represented, followed by binding, transporter activity, and antioxidant activity. Membrane and cell, with nearly identical DEG numbers, were the most highly represented GO terms in the cellular component category. In NZ1, although most of the main functions of DEGs were similar to those of Xu32, the gene numbers of the main functions were considerably less compared to those in Xu32.

To further investigate which cellular network could be regulated by K^+^-deficiency stress, KEGG pathway enrichment analysis was performed. The results of KEGG enrichment analysis of DEGs are shown in Fig. S2. Twelve selected metabolic pathways were analyzed. The three pathways of phenylpropanoid biosynthesis, the metabolic pathway, and glycolysis/gluconeogenesis were the common crucial enriched pathways in both Xu32 and NZ1. Secondary metabolites related to abiotic stress were also enriched in the two different genotypes. However, the pathway of carbon fixation in photosynthetic organisms was particularly enriched in Xu32, and not in NZ1, possibly resulting in higher photosynthetic efficiency in Xu32 than in NZ1 under K^+^-deficiency stress (Fig. 2).

### Functional annotation of common metabolic pathways in the two sweetpotato genotypes

**Fig. 6:**
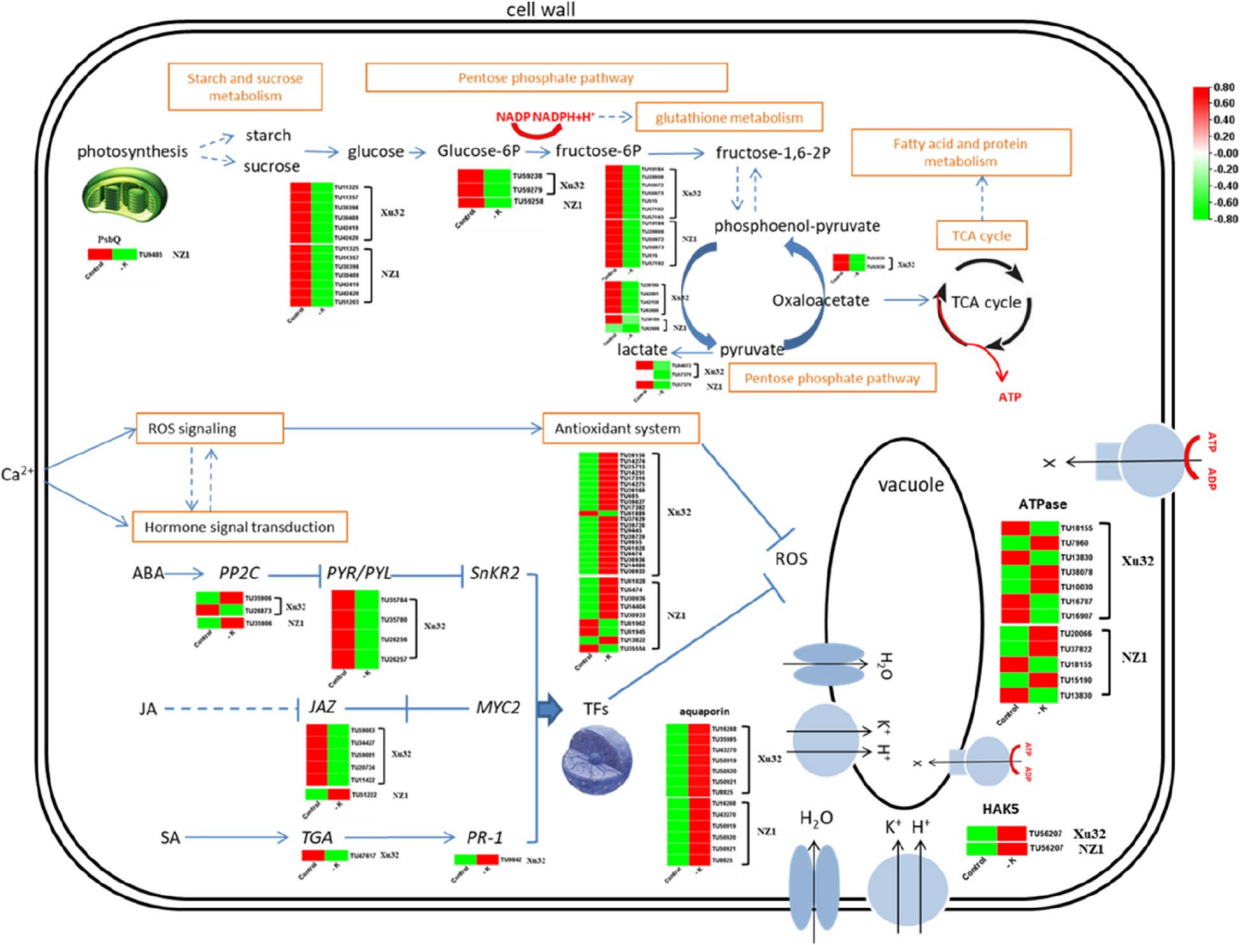
Transcriptional changes of genes responsible for K^+^-deficiency tolerance in sweetpotato cv. Xu32 and cv. NZ1. The heatmap shows the gene ID numbers and expression patterns of differentially expressed genes (DEGs) involved in photosynthesis, glucose metabolism (starch and sucrose metabolism, the pentose phosphate pathway, the hormone transduction pathway, or encoding transporter proteins and radical oxygen species (ROS)-scavenging enzymes in both sweetpotato cultivars. The associated metabolites are shown in orange boxes, and the blue arrows denote the upstream/downstream relationships in the K^+^-deficiency stress response pathway. For transporters, the black arrow direction denotes the transportation direction of K^+^, H^+^, H_2_O, and other ions

To determine the mechanism of low-K^+^ tolerance in sweetpotato, the common and distinct metabolic pathways between Xu32 and NZ1 plants were analyzed. According to GO functional annotation, 13 DEGs in Xu32 and 11 DEGs in NZ1 encoded transporters (Table 2; Fig. 6). Aquaporins play important roles in maintaining cell homeostasis under abiotic stress [21]. In the current study, six common genes (TU43270, TU50919, TU50920, TU50921, TU16268, and TU8825) encoding aquaporin were upregulated under K^+^-deficiency treatment in the two genotypes. The high-affinity K^+^ transporter (HAK) family, as the largest K^+^ transporter family, plays a major role in K^+^ acquisition under low external K^+^ contents in plants [22]. *IbHAK5* (TU56207) was the only identified K^+^ transporter DEG in the two sweetpotato genotypes. However, the relative transcript level of the gene was obviously higher in Xu32 than in NZ1 under normal and low-K^+^ stress conditions. Three DEGs encoding zinc transporters were only identified in Xu32 and were downregulated upon K^+^ starvation.

**Table 2:** Genes encoding transporter and ATPase differently expressed in Xu32 and NZ1 subjected to K^+^-deficiency

Primary classification	Secondary classification	Gene ID	Annotation	Fold change						Description
				Xu32 Control	Xu32 -K	log2(fc)	NZ1 Control	NZ1 -K	log2(fc)	
**Transporter**	**water transmembrane transporter activity**	TU35985	TIP1-2	79.03473	461.5444	2.545911	/	/	/	PREDICTED: probable aquaporin TIP1-2 [Ipomoea nil]
		TU43270		169.1897	698.7485	2.046131	224.348	562.7415	1.326734	tonoplast intrinsic protein [Ipomoea batatas]
		TU50919	TIP2-1	127.3291	400.4703	1.653133	116.7953	266.5569	1.190461	PREDICTED: aquaporin TIP2-1-like [Ipomoea nil]
		TU50920		66.14229	230.1577	1.798978	132.5772	320.5885	1.27389	PREDICTED: aquaporin TIP2-1-like [Ipomoea nil]
		TU50921		58.02291	205.9454	1.827567	54.90241	146.0359	1.411382	PREDICTED: aquaporin TIP2-1-like [Ipomoea nil]
		TU16268	NIP3-1	21.4401	71.20128	1.731591	11.10222	41.33501	1.896516	PREDICTED: probable aquaporin NIP5-1 [Ipomoea nil]
		TU8825		14.09597	98.18821	2.800267	13.88493	73.3475	2.401228	PREDICTED: probable aquaporin NIP5-1 [Ipomoea nil]
	**ion transmembrane transporter activity**	TU39231		82.28009	24.12548	-1.76999	/	/	/	zinc transporter [Dorcoceras hygrometricum]
		TU39232		85.0595	21.96933	-1.95298	/	/	/	zinc transporter [Dorcoceras hygrometricum]
		TU39233		67.37929	16.74666	-2.00843	/	/	/	zinc transporter [Dorcoceras hygrometricum]
		TU30580	AMT1-2	/	/	/	3.709936	23.63107	2.671219	PREDICTED: ammonium transporter 1 member 2-like [Ipomoea nil]
		TU45836	SUC2	/	/	/	0.001	13.14799	13.68255	PREDICTED: sucrose transport protein-like [Ipomoea nil]
		TU41372	MST4	48.56923	4.277517	-3.5052	/	/	/	PREDICTED: sugar transport protein 13 isoform X2 [Ipomoea nil]
		TU41377	STP13	278.893	32.17482	-3.11571	369.8621	103.4234	-1.83843	PREDICTED: sugar transport protein 13 isoform X2 [Ipomoea nil]
		TU56207	HAK5	16.59343	73.71624	2.15137	3.465779	36.0174	3.377442	PREDICTED: potassium transporter 5-like [Ipomoea nil]
	**anion transmembrane transporter activity**	TU53335	DTC	/	/	/	62.84858	132.6462	1.077631	PREDICTED: mitochondrial dicarboxylate/tricarboxylate transporter DTC [Ipomoea nil]
**ATPase**		TU18155	AATP1	77.85427	29.37918	-1.40598	/	/	/	PREDICTED: AAA-ATPase At3g28580-like [Ipomoea nil]
		TU7960		138.3336	294.233	1.088807	/	/	/	PREDICTED: AAA-ATPase ASD, mitochondrial-like [Ipomoea nil]
		TU13830	PMA4	280.884	133.7395	-1.07055	240.9331	115.2761	-1.06354	PREDICTED: plasma membrane ATPase 4 [Ipomoea nil]
		TU38078	ACA2	62.14979	162.2226	1.384154	/	/	/	PREDICTED: calcium-transporting ATPase 2, plasma membrane-type-like [Ipomoea nil]
		TU10030	VHA-a3	0.213928	14.68913	6.101477	/	/	/	PREDICTED: V-type proton ATPase subunit a3-like isoform X2 [Ipomoea nil]
		TU16787	ABCB15	60.9475	7.313475	-3.05894	/	/	/	PREDICTED: putative ABC transporter B family member 8 [Ipomoea nil]
		TU16907		18.61233	3.32571	-2.48452	/	/	/	PREDICTED: putative ABC transporter B family member 8 [Ipomoea nil]
		TU20066	PMA4	/	/	/	6.329191	68.18838	3.429433	PREDICTED: plasma membrane ATPase 4-like [Ipomoea nil]
		TU37822	PDR2	/	/	/	0.001	12.89335	13.65434	PREDICTED: probable manganese-transporting ATPase PDR2 [Ipomoea nil]
		TU18155	AATP1	/	/	/	50.63312	19.77166	-1.35665	PREDICTED: AAA-ATPase At3g28580-like [Ipomoea nil]
		TU15190	RECQL5	/	/	/	17.62922	51.66667	1.551265	PREDICTED: ATP-dependent DNA helicase Q-like 5 [Ipomoea nil]

The antioxidative defense system, which is composed of enzymatic antioxidant enzymes, contributes to removing the excess reactive oxygen species (ROS) produced under environmental stress [23]. In the present study, five common DEGs (TU61028, TU6474, TU30936, TU14404, and TU30933) encoding antioxidant enzymes were identified in both Xu32 and NZ1 (Table 3; Fig. 6). Additionally, a total of 16 unique DEGs (15 upregulated and one downregulated) encoding antioxidant enzymes were identified in Xu32, whereas only four unique DEGs (one upregulated and three downregulated) were identified in NZ1.

Most stress conditions are accompanied by changes in sugar distribution, metabolism, and transportation. Sugar is an important energy source that acts as a metabolic substrate as well as a signaling molecule between cells [24]. In the present study, 14, 35, 10, 8, and 2 DEGs in Xu32 were found to be involved in starch and sucrose metabolism, glycolysis/gluconeogenesis, the pentose phosphate pathway, pyruvate metabolism, and the TCA cycle, respectively, while there were 14, 19, 8, 3, and 1 corresponding DEGs in NZ1, respectively.

In brief, these key DEGs encoding transporters, or involved in the antioxidative defense system and sugar metabolism pathway, may contribute to K^+^-deficiency stress tolerance, but the different DEG numbers and expression intensity may give rise to different levels of tolerance to K^+^-deficiency stress between the two sweetpotato genotypes.

### Functional annotation of distinct metabolic pathways in the two sweetpotato genotypes

PsbQ proteins are found in the thylakoid lumen of chloroplasts and regulate photosystem II (PSII) activity via influencing several parameters of PSII function. In this study, a photosynthesis-related gene encoding PsbQ (TU9485) was detected and was found to be downregulated under K^+^-deficiency stress, with subsequent severe damage of the photosynthetic system in NZ1, while this gene was not differentially regulated in Xu32 (Table 4; Fig. 6).

ABA, SA, and JA are prominent stress hormones involved in the response to various stressful conditions. Importantly, after K^+^-deficiency stress treatment, an opposite change trend of the hormone content was observed between Xu32 and NZ1 (Fig. 3). To further determine how the three hormones acted in response to K^+^-deficiency stress, the present study evaluated the DEGs involved in the targeted hormone signal transmission. The ABA receptor family (PYR/PYL) and protein phosphatase 2 C (PP2C) are involved in the ABA-independent pathway response to various stresses [25]. In the present study, four downregulated genes encoding ABA receptor PYL (TU35784, TU35780, TU26256, and TU26257) and two genes encoding PP2C (one upregulated gene, TU35906, and one downregulated gene, TU26873) were found in Xu32 under K^+^-deficiency stress, whereas only one upregulated gene encoding PP2C (TU35906) was identified in NZ1 (Table 4; Fig. 6). Additionally, the TIFC/JAZ gene family represses the activity of transcription factors that promote the expression of JA-response genes [26]. A total of five genes encoding TIFC (TU59083, TU34427, TU59081, TU20724, and TU1422) were downregulated in Xu32, whereas only one gene encoding TIFC (TU51222) was upregulated in NZ1. Moreover, one gene encoding PR1 (TU9842), an SA-inducible marker gene, was upregulated only in Xu32 (Table 4; Fig. 6).

**Table 3:** Reactive oxygen species (ROS) metabolism-related DEGs in Xu32 and NZ1 subjected to K^+^-deficiency

Gene ID	Annotation	Fold change						Describtion
		Xu32 Control	Xu32 -K	log2(fc)	NZ1 Control	NZ1 -K	log2(fc)	
TU26156	PNC1	6.537692	70.76771	3.436238	/	/	/	PREDICTED: peroxidase P7-like [Ipomoea nil]
TU14274	PER72	29.49863	128.0755	2.118275	/	/	/	anionic peroxidase swpb1 [Ipomoea batatas]
TU25713	pod	6.323947	46.49229	2.878094	/	/	/	anionic peroxidase swpa5 [Ipomoea batatas]
TU14251	PER72	67.14962	199.8297	1.57332	/	/	/	anionic peroxidase swpb2 [Ipomoea batatas]
TU17319	GSVIVT00037159001	6.01901	53.38118	3.148733	/	/	/	PREDICTED: peroxidase 5-like [Ipomoea nil]
TU14275	PER72	16.84425	62.86478	1.899996	/	/	/	anionic peroxidase swpb1 [Ipomoea batatas]
TU26169	PNC1	6.415424	32.91183	2.35899	/	/	/	PREDICTED: cationic peroxidase 1-like [Ipomoea nil]
TU605	PER56	8.851761	54.20524	2.614396	/	/	/	PREDICTED: peroxidase 27-like [Ipomoea nil]
TU39627	PER21	15.4753	75.39201	2.284444	/	/	/	PREDICTED: peroxidase 21 [Ipomoea nil]
TU17382	GSVIVT00037159001	3.247527	27.9465	3.105254	/	/	/	PREDICTED: peroxidase 5-like [Ipomoea nil]
TU61889	PNC1	31.59673	9.841801	-1.68278	/	/	/	PREDICTED: cationic peroxidase 1-like [Ipomoea nil]
TU37629	GSVIVT00037159001	2.597305	20.29781	2.966236	/	/	/	PREDICTED: peroxidase 5-like [Ipomoea nil]
TU39728	POD1	6.528193	27.84688	2.09276	/	/	/	PREDICTED: lignin-forming anionic peroxidase-like [Ipomoea nil]
TU9445	TAP2	12.69672	47.32488	1.898143	/	/	/	anionic peroxidase swpa2 [Ipomoea batatas]
TU39729	PER5	2.385626	17.07065	2.839079	/	/	/	PREDICTED: lignin-forming anionic peroxidase-like [Ipomoea nil]
TU9955	PER10	22.64319	60.29657	1.412999	/	/	/	PREDICTED: peroxidase 10 [Ipomoea nil]
TU61028	PER42	25.90934	87.97132	1.763561	20.9765	77.08022	1.877587	PREDICTED: peroxidase 42 [Ipomoea nil]
TU6474	PER3	19.98768	83.61126	2.064586	6.936396	84.07768	3.599465	PREDICTED: peroxidase 3-like [Ipomoea nil]
TU30936	PER3	32.15942	101.2203	1.654186	23.26737	72.89263	1.647465	basic peroxidase swpb7 [Ipomoea batatas]
TU14404	PER27	22.00825	113.8169	2.370598	3.932238	35.16281	3.160628	anionic peroxidase swpa9 [Ipomoea batatas]
TU30933	PER27	10.02846	63.83819	2.67032	5.114932	67.4142	3.720266	PREDICTED: peroxidase 27-like [Ipomoea nil]
TU61962	PNC1	/	/	/	35.44055	5.689564	-2.63901	PREDICTED: cationic peroxidase 1-like [Ipomoea nil]
TU61945	PNC1	/	/	/	35.98188	4.966566	-2.85695	PREDICTED: cationic peroxidase 1-like [Ipomoea nil]
TU13822	PER16	/	/	/	1.400321	17.29478	3.626507	PREDICTED: peroxidase 16-like [Ipomoea nil]
TU35554	GSVIVT00023967001	/	/	/	63.48513	20.94653	-1.59971	PREDICTED: peroxidase 4-like isoform X5 [Ipomoea nil]

**Table 4:** Hormones-related and photosynthesis-related DEGs in Xu32 and NZ1 subjected to K^+^-deficinecy

Primary classification	Secondary classification	Gene ID	Annotation	Fold change						Description
				Xu32 Control	Xu32 -K	log2(fc)	NZ1 Control	NZ1 -K	log2(fc)	
Hormones	ABA	TU35784	PYL4	702.7875	153.4704	-2.19513	/	/	/	PREDICTED: abscisic acid receptor PYL4-like [Ipomoea nil]
		TU35780		1062.61	281.8771	-1.91447	/	/	/	PREDICTED: abscisic acid receptor PYL4-like [Ipomoea nil]
		TU26256		722.8516	276.8045	-1.38483	/	/	/	PREDICTED: abscisic acid receptor PYL4-like [Ipomoea nil]
		TU26257		169.7531	76.8755	-1.14284	/	/	/	PREDICTED: abscisic acid receptor PYL5-like [Ipomoea nil]
		TU35906	PP2CA	29.97801	77.18974	1.364504	39.51797	87.03188	1.139035	PREDICTED: protein phosphatase 2 C 37-like [Ipomoea nil]
		TU26873	PP2C27	18.05049	0.001	-14.1398	/	/	/	PREDICTED: probable protein phosphatase 2 C 27 isoform X1 [Ipomoea nil]
	JA	TU59083	JAZ/TIFY	50.96773	1.9747	-4.68988	/	/	/	PREDICTED: protein TIFY 9-like isoform X2 [Ipomoea nil]
		TU34427		203.0184	50.13162	-2.01782	/	/	/	PREDICTED: protein TIFY 10 A-like [Ipomoea nil]
		TU59081		170.7713	60.02072	-1.50853	/	/	/	PREDICTED: protein TIFY 9-like isoform X2 [Ipomoea nil]
		TU20724		268.9135	102.2277	-1.39536	/	/	/	PREDICTED: protein TIFY 9-like isoform X2 [Ipomoea nil]
		TU11422		498.2065	207.4792	-1.26378	/	/	/	JAZ6, partial [Ipomoea batatas]
		TU51222		/	/	/	312.5271	817.038	1.386422	JAZ1 [Ipomoea batatas]
	SA	TU47617	TGA	55.10021	6.477586	-3.08853	/	/	/	PREDICTED: TGACG-sequence-specific DNA-binding protein TGA-1 A-like isoform X2 [Ipomoea nil]
		TU9842	PR-1	14.43786	58.1443	2.00978	/	/	/	PREDICTED: pathogenesis-related leaf protein 4-like [Ipomoea nil]
Photosynthesis		TU9485	PsbQ	/	/	/	73.55619	23.29029	-1.65912	PREDICTED: oxygen-evolving enhancer protein 3 − 1, chloroplastic [Ipomoea nil]

Low potassium can regulate the expression of several stress-related genes. The expression levels of two genes encoding galactinol synthase (GOLS2, genes TU17507 and TU34960), one gene encoding heavy metal-associated isoprenylated plant proteins (HIPP26, the TU4051 gene), one gene encoding D-aminoacyl-tRNA deacylase (GEK1, the TUTU22143 gene), and one gene encoding basic endochitinase (CHIT1B, the TU14176 gene) were increased by K^+^ deficiency in both sweetpotato genotypes. However, the expression level of cytochrome P450s (CYP450, genes TU23888, TU59206, TU59207, and TU23870) and pheophorbide *a* oxygenase (PAO, genes TU56536 and TU56537) was uniquely induced by low potassium in Xu32, whereas ROSINA (RSI, gene TU47825), metallothionein (pKIWI, gene TU49279), and dual-specificity protein kinase splA (tag, gene TU58557) were uniquely induced in NZ1 (Table S3).

## Discussion

Potassium is an essential soil nutrient that ultimately affects yields, especially for tuber root crops. A series of adaptive biochemical and physiological responses have evolved in plants to cope with K^+^ deficiency. In the present study, the apparent differences of phenotype, physiological and biochemical characteristics, and resistance mechanisms after K^+^-deficiency stress treatment in two sweetpotato genotypes were observed. First, the phenotypic responses of Xu32 and NZ1 were compared. The results showed that Xu32 had a higher capability to absorb K^+^ than NZ1, with better growth performance, a higher Pn, and higher chlorophyll contents under low-potassium stress. In addition, RNA-seq analysis was used to reveal the molecular mechanism of sweetpotato in response to K^+^-deficiency stress.

The reduction of the potassium concentration and accumulation led to the decline of photosynthetic function in the two sweetpotato genotypes, thereby causing the source-sink relationship to become unbalanced, which resulted in dry weight loss. Low-K^+^ stress caused changes in sugar metabolism, including anabolism and catabolism. Environmental stresses, such as low or high temperature, osmotic stress, nutrient stress, and pests and diseases, can also trigger changes in sugar metabolism [24]. Photosynthesis is the main source of sugar. The distinct expression level of PsbQ between Xu32 and NZ1 may have contributed to the difference in the extent of Pn damage, causing the end product of photosynthetically produced sugar to differ greatly between the two genotypes. Low K^+^ also regulated the expression of genes related to starch and sucrose metabolism, such as sucrose synthase (SS and SUS), which may have decreased the activity of sucrose synthetase in sweetpotato. More downregulated genes were involved in sugar catabolism in Xu32 than in NZ1, including starch and sucrose metabolism, the glycolysis/gluconeogenesis pathway, the pentose phosphate pathway, pyruvate metabolism, and the citrate cycle, which may have been correlated with the different changes in soluble sugars between the two sweetpotato genotypes. Moreover, trehalose 6-phosphate (T6P) is a sugar molecule with signaling function that reports the current sucrose state and plays a critical role in plant responses to environmental stresses [27]. Trehalose synthesis is catalyzed by trehalose-6-phosphate synthase (TPS) and T6P. In the present study, the TPP expression level was highly upregulated in sweetpotato plants exposed to low-potassium stress, indicating that T6P may regulate plants subject to K^+^-deficiency. In brief, the changes in sugar allocation, metabolism, and transport under low-K^+^ stress contribute to low-K^+^ resistance in sweetpotato.

K^+^ transporters and K^+^ channels are important factors that contribute to K^+^ uptake and translocation. Our previous studies identified and analyzed the K^+^ transporter and channel gene families in sweetpotato and verified that *IbAKT1* and *IbHKT1* function in K^+^ absorption [8, 28]. HAK5, belonging to the high-affinity uptake system, is the only one capable of supplying K^+^ to the plant under very low external K^+^ conditions [29]. In the present study, *IbHAK5* was highly induced in the two sweetpotato genotypes under K^+^-deficient conditions. It was also found that some aquaporin genes were significantly upregulated under low-potassium stress. Aquaporins have an important regulatory function in water transport and the transport of other molecules across the cell membrane and through the intercellular compartment, thereby playing important roles in maintaining cell homeostasis under abiotic stress [30]. On the basis of phylogenetic distribution and subcellular occurrence, the following five subfamilies have been classified among the aquaporin isoforms: plasma membrane intrinsic proteins (PIPs), tonoplast intrinsic proteins (TIPs), nodulin-like proteins (NIPs), small basic intrinsic proteins (SIPs), and uncharacterized intrinsic proteins (XIPs) [31]. In the present study, most of the upregulated aquaporins were TIPs, implying that tonoplast alleviated the damage caused by low potassium stress through containing stored water and regulating the movement of water. In addition, the Ca^2+^ transporter Ca^2+^-ATPase, ABC transporters, and sucrose and sugar transporters were also involved in the K^+^-starvation resistance process, with changed transcription levels.

Plant endogenous hormones not only control plant growth and development under normal conditions but also mediate plant adaption to various environmental stresses [32]. ABA, as a key regulator of abiotic stress responses in plants, is significantly triggered by salt and drought stresses [33]. The ABA core signaling pathway largely relies on the expression of the ABA receptor PYR/PYL to mediate several rapid responses to complex environmental conditions [34]. In the present study, the DEGs encoding PYLs were only inhibited in Xu32, while they remained unchanged in NZ1, implying that low-potassium stress could activate the ABA signaling pathway in the low-potassium-tolerant genotype. SA and JA mediate the regulation and control of abiotic stress tolerance in plants, and the external application of JA and SA can enhance plant resistance [35, 36]. It has been reported that SA and JA may also play a role in the process of plant resistance against nutrient starvation stress, but the mechanism remains unclear [37]. The JA receptor COI1 can sense JA and bind to JAZ, after which JAZ is ubiquitinated and degraded, and downstream transcription factors or signal transduction proteins of JAZ are released, thus promoting the resistance response regulated by JA [38]. In the present study, the expression levels of JAZs decreased in Xu32, whereas they increased in NZ1, indicating that the JA signaling pathway was activated in Xu32 and that the accumulated JA may have allowed Xu32 to adapt to K^+^-starvation stress. Similarly, we inferred that SA signaling was activated in Xu32 but not in NZ1 because the key genes involved in SA signaling, such as transcription factor TGA and its downstream gene PR, were regulated only in Xu32. In addition, the contents of ABA, JA, and SA were determined, and the contents of these hormones exhibited opposite change trends in the two sweetpotato genotypes. Hormone receptors may have sensed the accumulated ABA, JA, and SA during low-K^+^ stress treatment and then activated the corresponding hormone pathways and downstream genes, thus decreasing the damage induced by low-K^+^ stress in Xu32. In contrast, long-term K^+^-deficiency treatment may have destroyed the normal growth of plants and resulted in cell death and decreased endogenous hormones in NZ1. The genes involved in early signal transduction pathways, such as Ca^2+^ signaling molecules and protein kinases, were not enriched in the present study, possibly also because of the experimental design of long-term K^+^-deficiency stress treatment.

Environmental stresses are usually accompanied by enhanced ROS production in plants, which induces oxidative stress and results in cellular damage and metabolic imbalance [39]. It is reported that ROS generation and scavenging pathways, as well as the expression of scavenging enzymes, change under various abiotic stress [40]. In the present study, the expression level of peroxidases increased more in Xu32 than in NZ1, implying that more ROS were scavenged in Xu32, thereby decreasing the oxidative stress under K^+^ deficiency to a greater degree.

Both common and distinct stress-related DEGs were found in Xu32 and NZ1, and the complex mechanism of tolerance to low potassium was further investigated. Plant adaptation to abiotic stress is significantly influenced by GolS, which is a regulatory enzyme that catalyzes the synthesis of raffinose family oligosaccharides (RFOs) [41]. The increased expression levels of two GolSs in both sweetpotato genotypes may contribute to strong low-potassium stress tolerance. In addition, HIPPs are involved in heavy metal stress tolerance and are induced in roots under excess Cd, Zn, Mn, and Cu stress [42]. In the present study, one HIPP was transcriptionally regulated in Xu32 and NZ1 under K^+^-starvation conditions, which may have also played a role in low-potassium stress tolerance. CYPs, as the largest enzyme family, are involved in NADPH- and/or O_2_-dependent hydroxylation reactions and are found in all domains of living organisms, including bacteria, plants, and mammals [43]. The transcription level of CYP450 shows different change trends in response to diverse stresses. For example, the expression of CtCYP71A1 in safflower was increased under drought stress, while it increased initially and subsequently decreased with ABA, GA3, and SA treatment [44]. In the present study, the decreased expression level of CYP450s in Xu32 but not in NZ1 under low-potassium stress may indicate that CYP450s are involved in the process of K^+^-deficiency stress response and may enhance the tolerance of Xu32.

## Conclusions

**Fig. 7:**
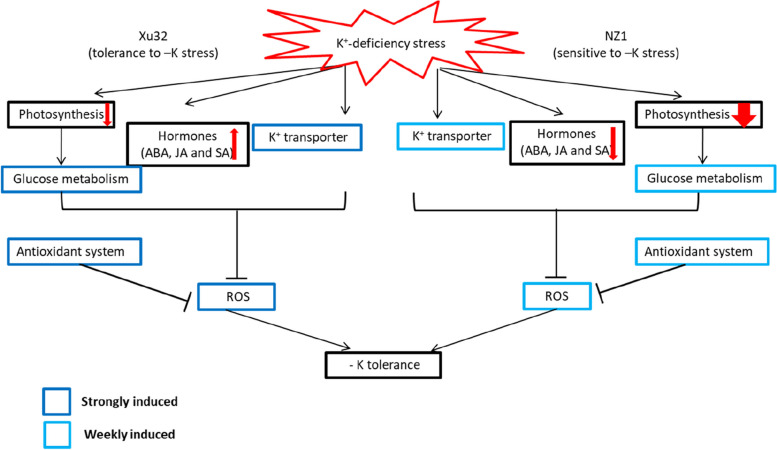
Hypothetical K^+^-deficiency stress response model for sweetpotato cv. Xu32 and cv. NZ1. In response to K+-deficiency stress, a more stable photosystem, higher numbers of differentially expressed genes (DEGs) involved in glucose metabolism, higher transcript levels of K^+^ transporters (*IbHAK5*), higher stress-related hormone contents, and stronger antioxidation reactions were induced in Xu32 than in NZ1, resulting in different levels of K^+^-deficiency tolerance. Dark and light blue boxes denote strongly and weakly induced metabolisms or expression genes by K^+^-deficiency stress, respectively. Arrows denote overall upstream/downstream relationships in the K^+^-deficiency stress response pathway. Thick and thin red arrows in photosynthesis indicate strongly and weakly decreased photosynthetic efficiency, respectively. The upstream and downstream direction of red-line arrows indicate increased and decreased contents of stress-related hormones (abscisic acid (ABA), salicylic acid (SA), and jasmonic acid (JA)), respectively

The two sweetpotato genotypes exhibited different physiological features and transcription levels under low-potassium stress. The common and distinct expression patterns between the two sweetpotato genotypes illustrate a complex mechanism in response to low potassium in sweetpotato. The greater number of DEGs identified in Xu32 than in NZ1 in response to K^+^ deficiency belonged to the process of photosynthesis, carbohydrate metabolism, ion transport, hormone signaling, stress-related genes, and antioxidant systems, possibly resulting in different levels of tolerance to low potassium (Figs. 6 and 7). Additionally, the findings of this study have provided some candidate genes that can be used in sweetpotato breeding programs aimed at improving low-potassium stress tolerance.

## Materials and methods

### Plant material and stress treatment

Two sweetpotato genotypes, Xu32 and NZ1, were cultivated in a greenhouse cultured hydroponically in a growth chamber under the following conditions: relative humidity, 50–70%; 12-h light/12-h dark photoperiod; temperatures, 30 °C days, 25 °C nights. Sweetpotato seedlings with consistent growth and similar features, including four leaves, a base stem diameter of 12–13 mm, a stem length of 20 ± 0.5 cm, and three internodes, were selected and transferred into light-proof boxes for hydroponic cultivation. The seedlings were cultured in water for 3 days for recovery and then transferred into modified Hoagland nutrient solution. The complete nutrient solution contained 20 mM K_2_SO_4_, 2 mM Ca (NO_3_)_2_.4H_2_O, 0.65 mM MgSO_4_.7H_2_O, 0.25 mM NaH_2_PO_4_.2H_2_O, 0.1 mM Fe-EDTA, 1 mM MnSO_4_, 1 mM ZnSO_4_.7H_2_O, 0.01 mM CuSO_4_.5H_2_O, 0.005 mM (NH_4_)_6_Mo_7_O_24_.4H_2_O, and 1 mM H_3_BO_3_, pH 6.0. For the low-K^+^ condition, the concentration of K_2_SO_4_ were changed to 1 mM, the remaining components were not altered. The sweetpotato seedlings were propagated and preserved by our laboratory. The whole plants were harvested at the 15th day of treatment for the determination of physiological performance and RNA-seq analysis.

### Determination of biomass, potassium content, and soluble sugar content in plants

For the determination of biomass, the seedlings of the two sweetpotato genotypes were harvested 15 days after beginning the K^+^-deficiency stress treatment. All plant samples were dried at 70℃ for 72 h until their weight remained constant, and the dry weight was measured.

The dried seedlings were ground and used for the measurement of K^+^ contents and soluble sugar contents. The soluble sugar content was determined using anthrone colorimetric analysis using the method reported by Ebell [45]. Each sample was taken from six different plants and mixed as a biological replicate. A total of three biological replicates were taken.

To analyze the K^+^ concentration, approximately 0.1 g dried samples were collected into chemical decomposition tube with 5 ml of concentrated sulfuric acid was then added for overnight. The following day, the samples were transferred and incinerated in a muffle furnace at 350 °C for 3 h. After the solution was cooled, several drops of 30% H_2_O_2_ were added until the solution become colorless. Flame photometry (PFP7; Jenway, UK) was used for determining K^+^ concentration with an exponential calibration curve drawn by 100, 50, 25, and 12.5 ppm potassium standard. A total of three biological replicates were taken.

### Analysis of photosynthetic activity

The net photosynthetic rate (Pn) was measured using a portable photosynthesis system (LI-6400 XT, LI-COR, Inc, Lincoln, NE, USA) at 12:00–2:00 pm on the 15th day of treatment. 2 g fresh leaves (the third to fifth) were cut into pieces and put into 50 ml centrifuge tubes with 2o ml 80% ethanol solution. Then, the centrifuge tubes were soaked in a dark place for overnight and chlorophyll content (chlorophyll A and chlorophyll B) were determined by a UV-visible spectrophotometer at 665 and 649 nm, respectively.

### Measurement of K^+^ fluxes in the roots

Sweetpotato root segments with 2-cm apices were used for K^+^ flux measurement with a noninvasive microtest system (NMT, NMT-100-SIM-YG, Younger USA LLC, Amherst, MA, USA). The measuring solution contained 0.1 mM NaCl, 0.1 mM MgCl_2_, 0.1 mM CaCl_2_, and 0.5 mM KCl. The steady K^+^ flux was recorded for 10 min in the meristem, elongation, and mature root zones.

### Hormone content measurement

Fresh sweetpotato samples were harvested, immediately frozen in liquid nitrogen, and extracted with methanol/water/formic acid (15:4:1, V/V/V). The combined extracts were reconstituted in 80% methanol (V/V) for liquid chromatography-mass spectrometry analysis. A total of 13 materials classified into abscisic acid (ABA), jasmonic acid (JA), and salicylic acid (SA) were collected via ultra-performance liquid chromatography (ExionLC™ AD, MASS, USA) and tandem mass spectrometry (QTRAP® 6500+, MASS, USA), and analysis was performed using Analyst ® 1.6.3 software (AB SCIEX™, MASS, USA).

### Total RNA extraction and cDNA library construction

The RNA from two different potassium concentration level treatments of two sweetpotato cultivars was extracted and monitored on 1% agarose gels for primary detection. The RNA purity (OD260/280 and OD260/230) and integrity were measured using the NanoPhotometer® spectrophotometer (IMPLEN, CA, USA). Sequencing libraries were generated using the NEBNext® Ultra™ RNA Library Prep Kit for Illumina® (NEB, California, USA), and the library quality was assessed on the Agilent Bioanalyzer 2100 system(Agilent, California, USA).

### Transcriptome sequencing and assembly

The library preparations were sequenced on the Illumina Hiseq 2000 platform according to the manufacturer’s instructions (Illumina, San Diego, CA, USA), and paired-end reads were generated. All raw sequence reads were deposited in the NCBI Sequence Read Archive (SRA) under accession number PRJAN1013090. After removing adapters and filtering low-quality reads from the raw data, high-quality clean reads were mapped to the sweetpotato reference genome sequence using Tophat2.0 software.

### Identification and functional annotation of differentially expressed genes (DEGs)

The DEG libraries prepared from samples of the potassium deficiency treatment and normal potassium treatment in two sweetpotato cultivars, namely the Xu32 Control, Xu32 -K, NZ1 Control, and NZ1 -K treatments, were constructed and sequenced. To estimate the gene expression levels, clean data were mapped back onto the assembled transcriptome using RSEM, and then, the read count for each gene was obtained from the mapping results and normalized using the reads per kilobase per million reads (RPKM) method. Differential expression analysis of two groups was performed using the DESeq R package (1.10.1) [46]. Gene Ontology (GO) enrichment analysis of the DEGs was implemented using the Goseq R packages based on Wallenius’ noncentral hypergeometric distribution [47], and Kyoto Encyclopedia of Genes and Genomes (KEGG) enrichment analysis was conducted to determine the statistical enrichment of DEGs in KEGG pathways using KOBAS (www.kegg.jp/kegg/kegg1.html) [48].

### Quantitative real-time polymerase chain reaction (qRT-PCR) analysis

Ten randomly selected common DEGs in Xu32 and NZ1 were subjected to qRT-PCR. During this procedure, 1 µg of total RNA was transcribed into cDNA using the TIANScriptIIRT Kit (TIANGEN, Beijing, China), and qRT-PCR was performed using the OneStep Real-Time System (Applied Biosystems, Foster City, California, USA) according to the manufacturer’s instructions. The reference gene Actin was used for normalization, and three independent biological replicates were performed for each sample. The comparative CT method (2-^△△^CT method) was used to analyze the gene expression levels. The specific primers used are listed in Table S5.

### Statistical analysis

The data were analyzed using the SPSS software program (SPSS Statistics v. 20.0, Chicago, IL, USA), and the results were presented as the sample means ± SD (*n* = 3). Statistical analysis was conducted using one-way analysis of variance (ANOVA), followed by Ducan’s test at a significance level of *P* < 0.05.

### Supplementary Information

**Additional file 1: Figure S1.** Total soluble sugars contents in two different K^+^-sensitive sweetpotato cultivars Xu32 and NZ1 under normal (Control) and K^+^-deficient conditions (-K) for two weeks. Date are means ± SE (*n*=3) and there is no significant difference between mean values of - K and control. **Figure S2.** Transcript levels of 12 randomly selected common DEGs in both cv. Xu32 and cv. NZ1 by qRT-PCR analysis. The columns represent relative expression obtained by qRT-PCR, and solid lines represent relative expression obtained by RNA-seq. Date are means ± SE (*n*=3). Primers used for qRT-PCR are listed in Table S5. A**Figure S3.** Gene ontology (GO) classification of DEGs in sweetpotato plants under K^+^-deficiency conditions. The enriched biological process, cellular component and molecular function GO terms of DEGs in cv. Xu32 (A) and in cv. NZ1(B). **Figure S4.** KEGG enrichment of DEGs in sweetpotato plants under K^+^-deficiency conditions. The top 20 enrichment KEGG pathway of DEGs in cv. Xu32 (A) and in cv. NZ1 (B).

**Additional file 2: Table S1.** GO classification of DEGs in Xu32 and NZ1 plants under K+-deficiency stress. **Table S2.** KEGG enrichment of DEGs in Xu32 and NZ1 plants under K+-deficiency stress. **Table S3.** Glucorse metabolism-related DEGs in Xu32 and NZ1 subjected to K+-deficinecy. **Table S4.** Stress-related DEGs in Xu32 and NZ1 subjected to K+-deficinecy. **Table S5.** Primers for qRT-PCR analysis used in this study.

## Data Availability

The RNA-seq datasets analysed during the current study are available in the NCBI repository, accession numbers: PRJNA1013090.
