# Supplementary material for: Comparative physiological and transcriptome analysis between potassium-deficiency tolerant and sensitive sweetpotato genotypes in response to potassium-deficiency stress
Source: BMC Genomics. 2024 Jan 15;25:61. doi: 10.1186/s12864-023-09939-5 (PMC10789036; doi:10.1186/s12864-023-09939-5)
Supplement: Supplementary file 1 — Additional file 1: Figure S1. Total soluble sugars contents in two different K+-sensitive sweetpotato cultivars Xu32 and NZ1 under normal (Control) and K+-deficient conditions (-K) for two weeks. Date are means ± SE (n=3) and there is no significant difference between mean values of - K and control. Figure S2. Transcript levels of 12 randomly selected common DEGs in both cv. Xu32 and cv. NZ1 by qRT-PCR analysis. The columns represent relative expression obtained by qRT-PCR, and solid lines represent relative expression obtained by RNA-seq. Date are means ± SE (n=3). Primers used for qRT-PCR are listed in Table S5. AFigure S3. Gene ontology (GO) classification of DEGs in sweetpotato plants under K+-deficiency conditions. The enriched biological process, cellular component and molecular function GO terms of DEGs in cv. Xu32 (A) and in cv. NZ1(B). Figure S4. KEGG enrichment of DEGs in sweetpotato plants under K+-deficiency conditions. The top 20 enrichment KEGG pathway of DEGs in cv. Xu32 (A) and in cv. NZ1 (B). [file 12864_2023_9939_MOESM1_ESM.docx]

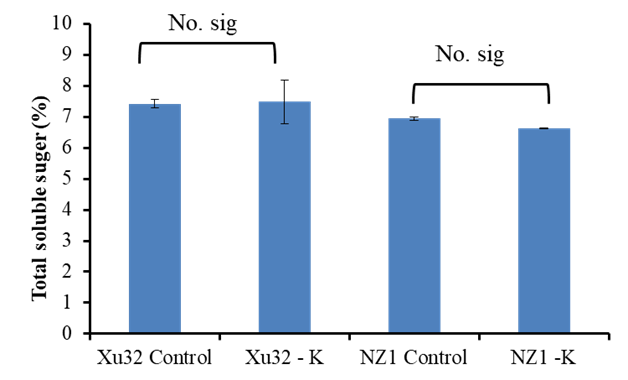


Figure S1. Total soluble sugars contents in two different K^+^-sensitive sweetpotato cultivars Xu32 and NZ1 under normal (Control) and K^+^-deficient conditions (-K) for two weeks. Date are means ± SE (n=3) and there is no significant difference between mean values of - K and control.


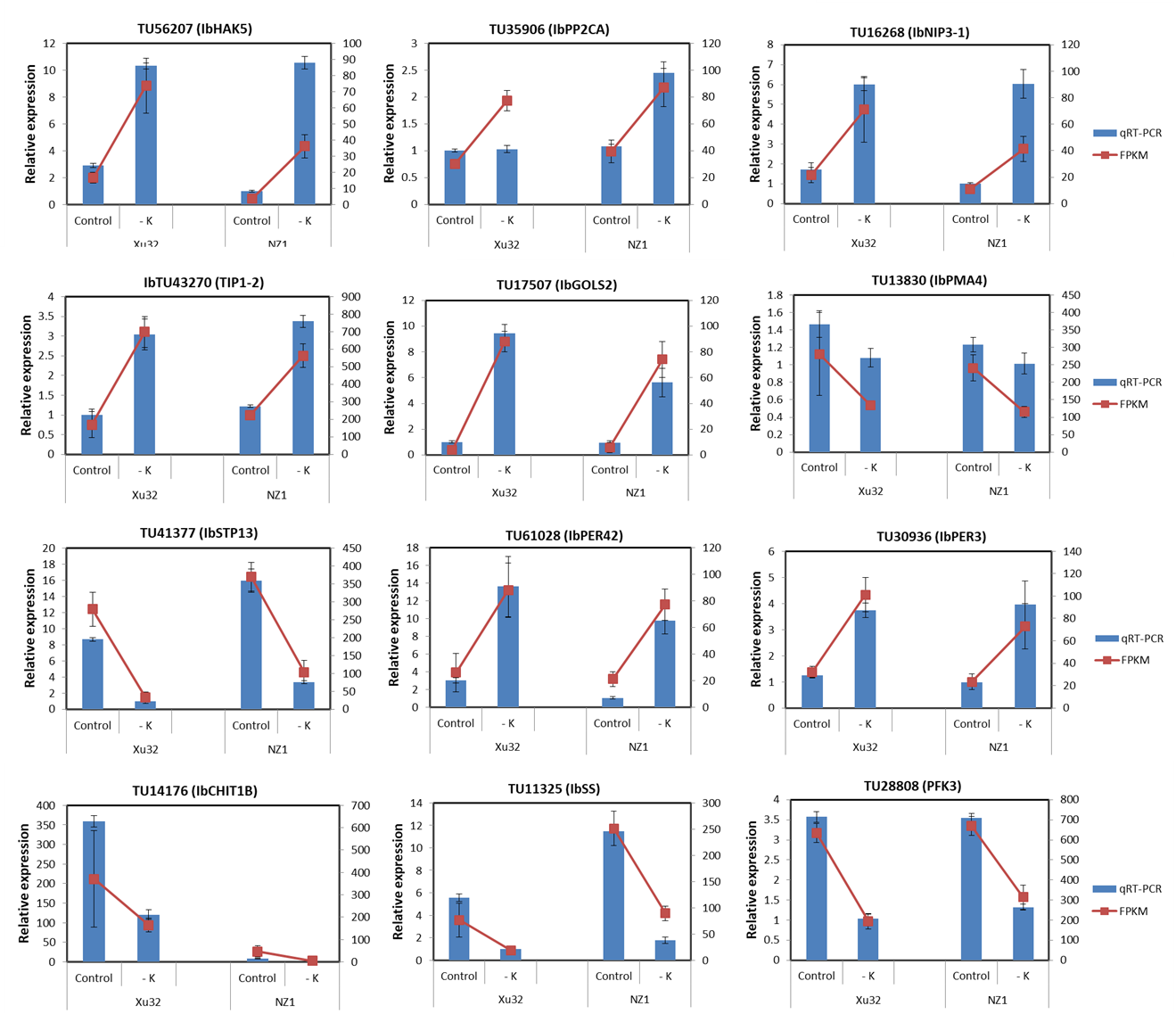


Figure S2. Transcript levels of 12 randomly selected common DEGs in both cv. Xu32 and cv. NZ1 by qRT-PCR analysis. The columns represent relative expression obtained by qRT-PCR, and solid lines represent relative expression obtained by RNA-seq. Date are means ± SE (n=3). Primers used for qRT-PCR are listed in Table S5.


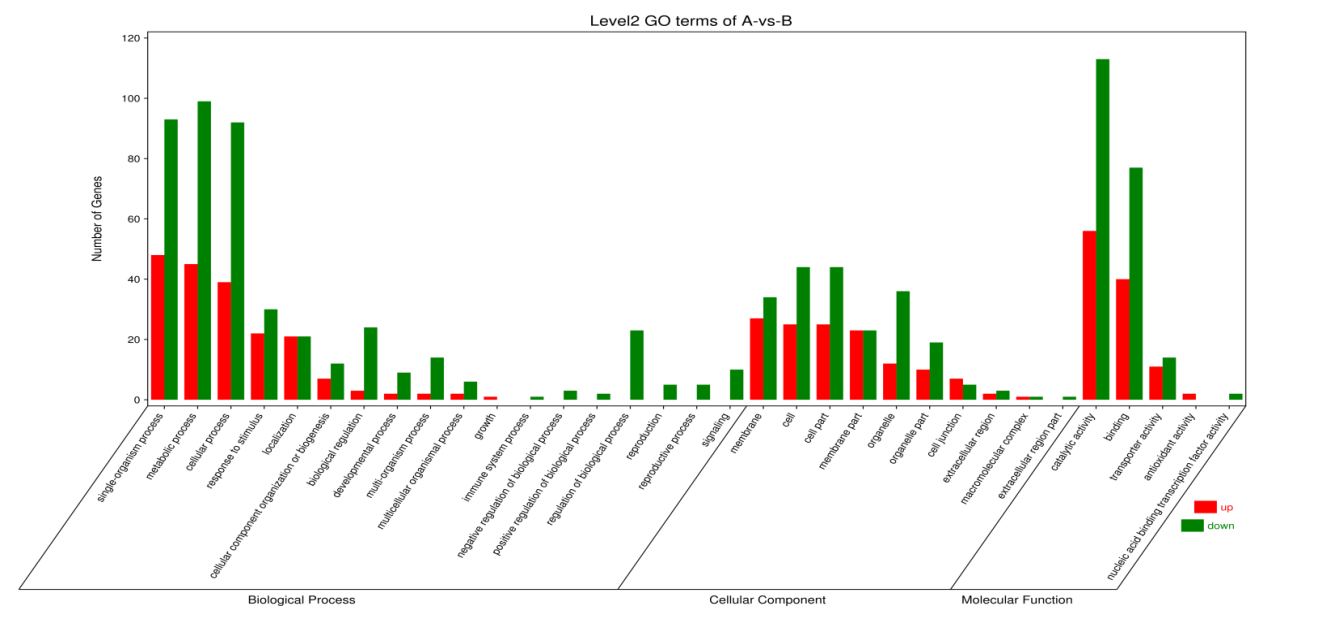

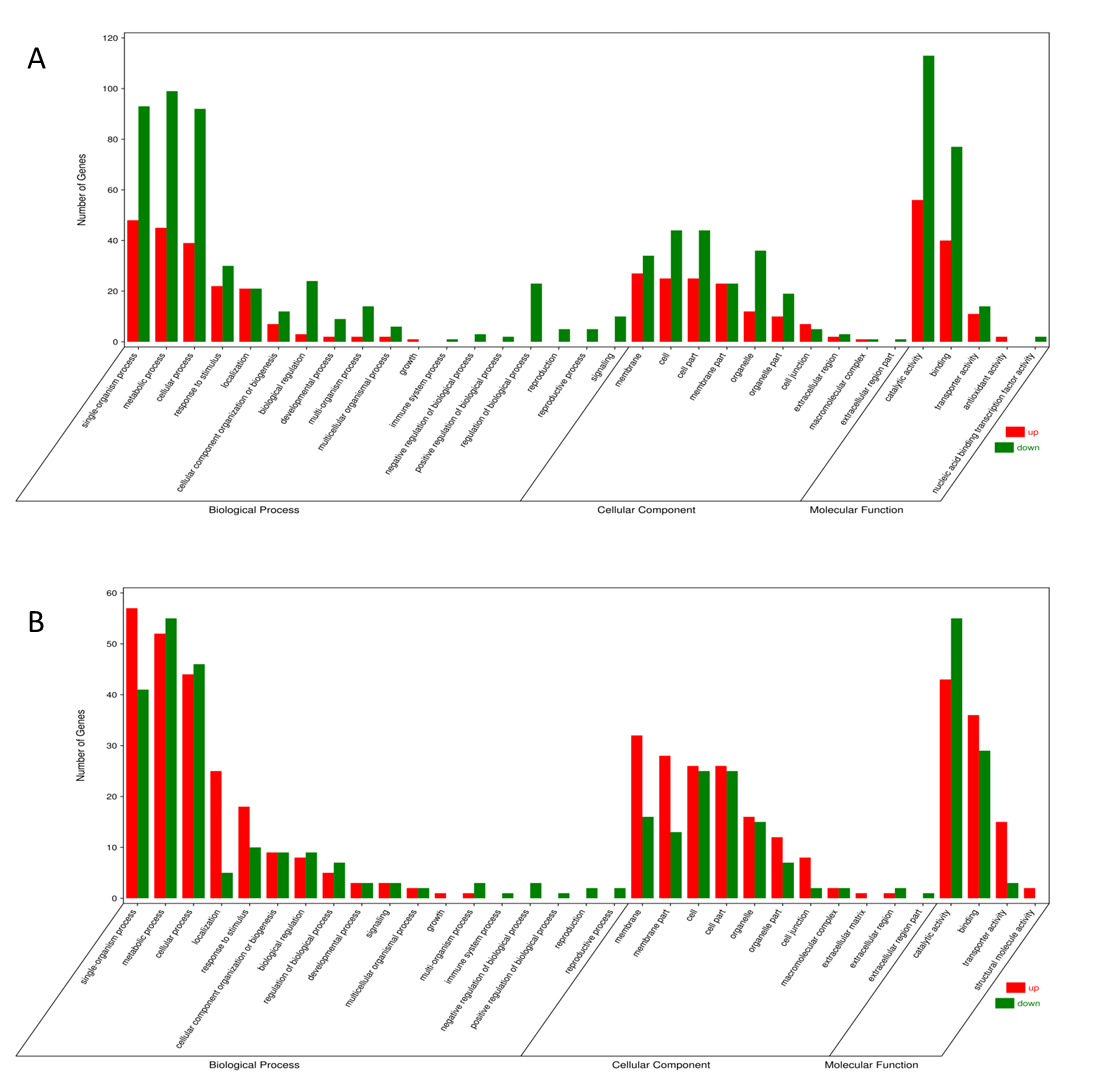


A

B

Figure S3. Gene ontology (GO) classification of DEGs in sweetpotato plants under K^+^-deficiency conditions. The enriched biological process, cellular component and molecular function GO terms of DEGs in cv. Xu32 (A) and in cv. NZ1(B).


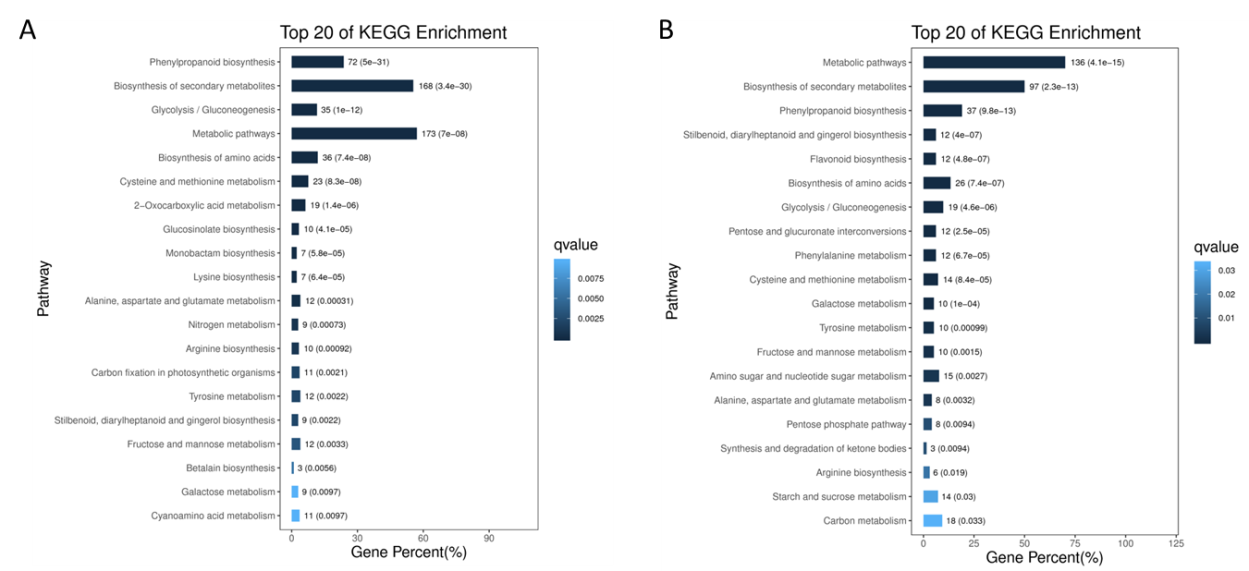


Figure S4. KEGG enrichment of DEGs in sweetpotato plants under K^+^-deficiency conditions. The top 20 enrichment KEGG pathway of DEGs in cv. Xu32 (A) and in cv. NZ1 (B).
